# Association between Fatty Acid Composition, Cryotolerance and Fertility Competence of Progressively Motile Bovine Spermatozoa

**DOI:** 10.3390/ani11102948

**Published:** 2021-10-13

**Authors:** Tanya Kogan, Dana Grossman Dahan, Ronit Laor, Nurit Argov-Argaman, Yoel Zeron, Alisa Komsky-Elbaz, Dorit Kalo, Zvi Roth

**Affiliations:** 1 Robert H. Smith Faculty of Agriculture, Food and Environment, The Hebrew University, Rehovot 76100, Israel; nushgros@gmail.com (D.G.D.); argov.nurit@mail.huji.ac.il (N.A.-A.); alisakomsky@yahoo.com (A.K.-E.); dorit.kalo@mail.huji.ac.il (D.K.); 2 Sion Artificial Insemination Center, Gedera 7057102, Israel; ronit@sion-israel.com (R.L.); yoel@sion-israel.com (Y.Z.); 3 Animal Sperm Research Center (ASRC), Robert H. Smith Faculty of Agriculture, Food and Environment, The Hebrew University, Rehovot 76100, Israel

**Keywords:** progressive motility, spermatozoa, semen cryopreservation, fatty acid composition, fertility, conception rate, freeze-thawing

## Abstract

**Simple Summary:**

Spermatozoa move forward in the female reproductive tract in a linear pattern defined as progressive motility (PM). PM is associated with fertilization competence and used as a parameter to evaluate the spermatozoa’s quality. The livestock-breeding industry is based on artificial insemination with cryopreserved spermatozoa, making the relationship between PM and cryosurvival important. This study explores the association between PM and spermatozoa’s quality, cryotolerance and fertilization competence. The number of progressively motile spermatozoa within a standard insemination straw positively correlates with conception rate, making progressively motile spermatozoa’s survival during cryopreservation a potentially relevant parameter for spermatozoa evaluation.

**Abstract:**

An association between progressive motility (PM) and spermatozoa fertility competence has been suggested. However, the mechanism that underlies PM is not clear enough. We examined physiological characteristics and fatty acid composition of fresh spermatozoa with high and low PM. Additional analysis of fatty acid composition and structural characteristics was performed on spermatozoa samples with high and low progressively motile spermatozoa’s survival (PMSS), i.e., the ratio between the proportion of progressively motile spermatozoa after and before cryopreservation. Finally, a fertility field trial was conducted to examine the association between the number of PM spermatozoa within the insemination straw post thawing and conception rate. Analysis of fresh spermatozoa revealed a higher omega-6 to omega-3 ratio in ejaculates with low PM relative to those with high PM (*p* < 0.01). The proportion of polyunsaturated fatty acids was higher in low-PMSS fresh samples (*p* < 0.05) relative to their high-PMSS counterparts. Fresh samples with high-PMSS expressed a higher mitochondrial membrane potential (*p* < 0.05) and a higher proportion of viable cells that expressed reactive oxygen species (ROS; *p* < 0.05). Post-thawing evaluation revealed a reduced proportion of progressively motile sperm, with a prominent effect in samples with high PM relative to low PM, defined before freezing (*p* < 0.01). No differences in spermatozoa mitochondrial membrane potential or ROS level were found post-thawing. A fertility study revealed a positive correlation between the number of progressively motile spermatozoa within a standard insemination straw and conception rate (*p* < 0.05). Considering these, the bull PMSS is suggested to be taken into account at the time of straw preparation.

## 1. Introduction

Progressive motility (PM) is the linear pattern of movement by which spermatozoa move forward in the female reproductive tract to meet the oocyte [1]. PM has been associated with fertilization competence, and is therefore widely used for sperm quality assessment [2,3]. In humans, PM serves as a reliable predictor of fertilization ability [4,5]. In equines, spermatozoa’s PM is positively correlated with fertility [6]. In buffalo bulls, the PM of spermatozoa post-thawing is associated with *in-vivo* fertility during the low-breeding season [7]. Interestingly, *in-vitro* fertilization with spermatozoa collected from crossbred bulls [8] or Holstein–Friesian bulls [9] was positively correlated with PM. In particular, the proportion of cleaved embryos and those developing to the blastocyst stage was higher when *in-vitro* fertilization was performed with high vs. low progressively motile spermatozoa. 

In dairy herds, the most common method used in reproductive management is artificial insemination (AI) with cryopreserved spermatozoa. Therefore, the relationship between PM and cryosurvival is highly important. Freezing and thawing procedures are characterized by osmotic stress, extracellular and intracellular ice-crystal formation, and production of reactive oxygen species (ROS) [10]. In addition, the toxicity of the cryoprotectants used during the process can impair the spermatozoa’s DNA, acrosome integrity, mitochondrial functionality, and motility [11,12].

The cryopreservation process has been reported to have a marked effect on the spermatozoa’s plasma membrane, expressed as altered permeability and fluidity [13,14]. In animals, the plasma membrane is composed of proteins and lipids, mostly glycerophospholipids and cholesterol molecules. The plasma membrane lipid composition, in particular the proportion of phospholipids, has a significant effect on spermatozoa’s function and motility, as reported for boars [15], dogs [16], and bulls [17]. In the latter, motility and PM of fresh spermatozoa were reported to correlate with monounsaturated fatty acid (MUFA) concentration and the omega-6:omega-3 ratio in the plasma membrane [17]. In our previous work, we reported on seasonal changes in membrane lipid composition in the spermatozoa’s tail compartment which were associated with reduced spermatozoa’s motility, PM and velocity during the summer [17]. Taken together, these findings reinforce the assumption that membrane lipid composition is a dominant cellular feature underlying spermatozoa’s function.

Here we hypothesize that spermatozoa with high PM differ in their membrane lipid composition from those with low PM, and therefore also differ in their ability to preserve PM following the cryopreservation process. Accordingly, our goal was to characterize the membrane status of spermatozoa with high and low PM, and high and low progressively motile spermatozoa’s survival (PMSS) during cryopreservation. We examined spermatozoa’s fatty acid composition and the integrity of the plasma membrane, mitochondrial membrane potential and spermatozoa’s oxidation status. In addition, a fertility study was conducted to establish a link between PM and fertilization competence. Exploring the associations between PM functionality, cryotolerance and fertilization competence is highly important for the preparation of straws used in intensive reproductive management.

## 2. Materials and Methods

### 2.1. Experimental Design

**Figure 1:**
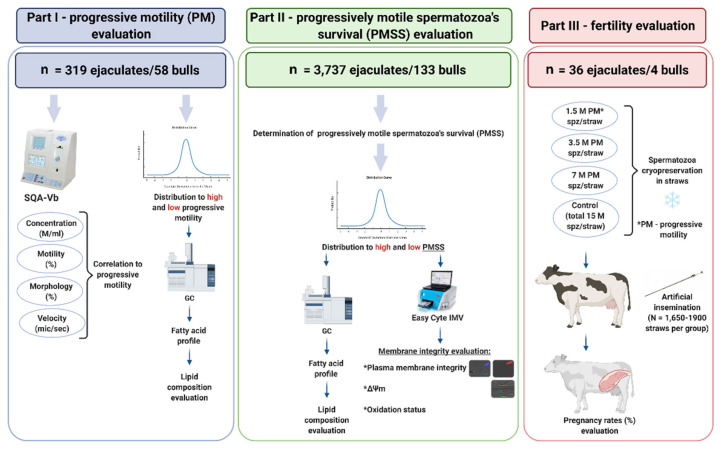
Schematic illustration of the experimental design. The study consisted of three parts: (1) a retrospective analysis of Sion Ltd.’s database to examine correlations between PM and physiological parameters. High-PM and low-PM thresholds were defined and the fatty acid composition were analyzed by GC; (2) based on Sion Ltd.’s database, the parameter of progressively motile spermatozoa’s survival (PMSS) was established, high-PMSS and low-PMSS thresholds were defined, and membrane fatty acid composition (GC) and integrity (FC) were analyzed; (3) a fertility study was conducted in which AI was performed with straws containing 15 M spermatozoa in total (control) or 1.5 M, 3.5 M or 7 M progressively motile spermatozoa per straw (PM spz/straw) after thawing.

The study included three parts, as presented in Figure 1.

#### 2.1.1. Part I: Spermatozoa Progressive Motility

**Figure 2:**
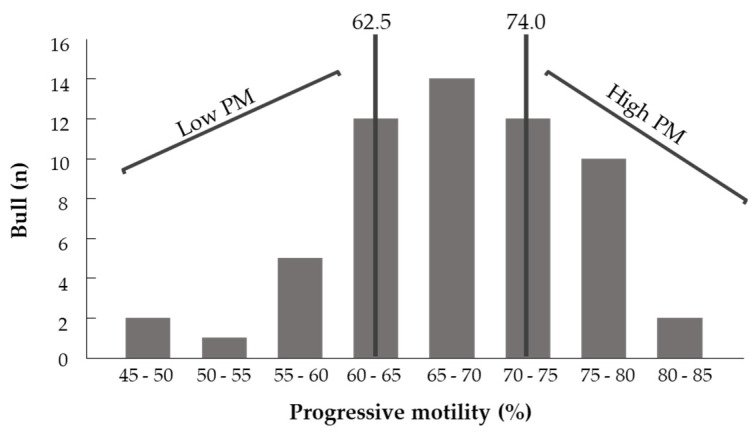
Distribution of bulls according to the proportion of progressively motile spermatozoa. Ejaculates with values higher than 74.0% (25% of the bull population with the highest PM) were defined as high PM and ejaculates with values lower than 62.5% (25% of the bull population with the lowest PM) were defined as low PM.

As a first step, a retrospective analysis was performed to examine the correlation between fresh spermatozoa’s PM and physiological parameters evaluated by a computerized sperm-quality analyzer calibrated for bull semen (SQA-Vb; Medical Electronic Systems, Caesarea, Israel). The data were taken from Sion Ltd.’s dataset and included 319 ejaculates collected from 58 bulls that were active at the time. A value higher than 74.0% was defined as high PM, and lower than 62.5% was defined as low PM (25% of the bull population with the highest and lowest PM, respectively) (Figure 2). Based on this, ejaculates with high and low PM (*n* = 9 and 6, respectively) were collected and analyzed for fatty acid composition by gas chromatography (GC).

#### 2.1.2. Part II: Progressively Motile Spermatozoa’s Survival

To examine the effect of cryopreservation on PM, a retrospective analysis was performed on an additional Sion Ltd. dataset which included 3737 ejaculates collected from 133 bulls. The decrease in percentage of motile and progressively motile spermatozoa due to the freeze–thaw process was calculated (∆ percent units). PMSS, i.e., the ratio between the proportions of progressively motile spermatozoa after freeze–thawing and in the fresh sample, was defined. A PMSS distribution curve was constructed based on 1363 ejaculates collected from 62 working bulls. A value higher than 68.1% was defined as high PMSS, and lower than 54.6% was defined as low PMSS (25% of the bull population with the highest and lowest PMSS, respectively). Based on this, fresh ejaculates with high PMSS (*n* = 21) and low PMSS (*n* = 20) were collected for GC analysis to characterize their fatty acid composition. In addition, fresh high PMSS (*n* = 21) and low PMSS (*n* = 24) and cryopreserved high PMSS (*n* = 19) and low PMSS (*n* = 19) ejaculates were examined for plasma membrane integrity, mitochondrial membrane potential and oxidation level by flow cytometry (FC).

#### 2.1.3. Part III: Fertility Evaluation

Analysis of Sion Ltd.’s sperm bank revealed a wide variation in the number of spermatozoa: between 1 M and 12 M spermatozoa per straw post-thawing. Based on this information, a field trial was conducted to evaluate the impact of the number of progressively motile spermatozoa on the pregnancy rate. The experiment involved 36 ejaculates from four fertile Israeli Holstein working bulls. Each ejaculate was divided into four experimental groups: a control group with 15 M total spermatozoa/straw post-thaw (i.e., the standard dilution used at Sion Ltd.) and three treatment groups with 1.5, 3.5 and 7 M progressively motile spermatozoa/straw post-thaw. Fresh ejaculate was diluted with AndroMed Extender using *B-Sperm^TM^* dosing software (Medical Electronic Systems, Caesarea, Israel), which determines an individual dilution factor by taking into account the decline in PM due to cryopreservation. The actual SQA-Vb measured post-thaw mean values were 13.4 M total spermatozoa with 3.7 M progressively motile spermatozoa per straw for the control group and 1.5, 3.0 and 6.0 M progressively motile spermatozoa per straw for the treatment groups, respectively. For each group, about 1650 to 1900 AIs were performed by professional inseminators at Sion Ltd. Pregnancy was confirmed by the farm veterinarian (via palpation) 42 days post insemination. Conception rate was calculated for each group as the number of pregnancies per total number of inseminations. The insemination procedure and pregnancy testing were performed blind.

### 2.2. Semen Collection and Processing

For all experiments, semen was collected from Holstein–Friesian bulls at Sion Ltd., the Israeli company for AI and breeding (Gedera, Israel). Bulls were mounted on a live teaser and semen was collected into a disposable tube using a heated (38 °C) sterile artificial vagina. Physiological characteristics of fresh and frozen spermatozoa were evaluated by computerized sperm-quality analyzer calibrated for bull semen (SQA-Vb; Medical Electronic Systems, Caesarea, Israel). SQA-Vb is an automated analytical device which makes use of electro-optic reading, video microscopy and software algorithms to perform an analysis of bull semen [18]. Semen samples were prepared and inserted into testing capillaries according to the manufacturer’s instructions, and then placed into the prewarmed SQA chamber for evaluation. The examined parameters were concentration ([M]/ml), motility (%), PM (%) and velocity (µm/s). Semen was diluted (AndroMed Extender, Minitube GmbH, Tiefenbach, Germany) using B-Sperm^TM^ dosing software (Medical Electronic Systems, Caesarea, Israel) to achieve a predetermined number of total or progressively motile spermatozoa post-thawing (Sion Ltd.’s protocol). Thereafter, diluted ejaculate was frozen in a DigitCool freezer (IMV Technologies, L’Aigle, France) at the following rate: −15 °C/min from 5 °C to −10 °C; and −40 °C/min from −10 °C to −140 °C, followed by storage in liquid nitrogen (−196 °C).

### 2.3. Lipid Extraction and Fatty Acid Analysis

Cold lipid extraction was performed using the Folch method followed by methylation to form fatty acid methyl esters (FAMEs) as previously reported [19]. FAME separation, identification, and quantification were performed in a 6890 N gas chromatograph (Agilent Technologies, Santa Clara, CA, USA) equipped with a flame ionization detector and fused silica capillary column (DB-23, 60 m × 0.25 mm ID, 0.25 µm film, Agilent Technologies, Santa Clara, CA, USA) under the following conditions: the oven temperature was programmed from 170 °C to 215 °C at a rate of 2.75 °C/min, from 215 °C to 250 °C at a rate of 40 °C/min, and held at 250 °C for 5 min. Peak identification was based on relative retention times of two external standards (FAME mix C4–C24 and FAME mix C16–C22). Relative concentrations of fatty acids were determined as molar percentage (mol %) of total fatty acids within each sample. Fatty acids were assigned to saturated fatty acids (SFAs), monounsaturated (MUFAs) and polyunsaturated fatty acids (PUFAs) according to their chemical structure. The PUFAs were additionally divided into omega-3 and omega-6 fatty acids.

### 2.4. Flow Cytometry

Flow cytometry tests were performed on fresh and frozen samples using the Guava EasyCyte microcapillary flow cytometer with CytoSoft software (Guava Technologies Inc., Hayward, CA, USA; distributed by IMV Technologies). The device detects particle-emission properties with three photomultiplier tubes (green: 525/30 nm, yellow: 583/26 nm, and red: 655/50 nm) and accommodated optical filters and splitters. Fresh spermatozoa were diluted with NKM buffer prewarmed to 37 °C (110 mM NaCl, 5 mM KCl, 20 mM MOPS [3-N-morphilino propane sulfonic acid; pH 7.4]) to obtain a concentration of 45 [M]/mL. Cryopreserved spermatozoa (2 × 0.225-mL French straws/sample) were thawed in distilled water (37 °C) for 30 s, placed in an Eppendorf tube and stored at room temperature. Each analysis was performed on 5000 spermatozoa. Plasma membrane integrity, mitochondrial membrane potential and the spermatozoa’s oxidation level were assessed with lyophilized fluorochrome-containing flow cytometry kits (EasyKit; IMV Technologies, L’Aigle, France) as previously described [20,21].

EasyKit 1 Viability and Concentration (ref. 024708; IMV Technologies) combines two fluorescent dyes to assess live and dead cells. The green dye stains the nuclei of living cells. The red dye penetrates damaged membranes, thus staining dead or moribund cells. Spermatozoa that display both green and red are categorized as dead. 

EasyKit 2 Mitochondrial Activity (ref. 024864; IMV Technologies) contains a lipophilic dye that penetrates and accumulates in the mitochondria. Spermatozoa with active (polarized) mitochondria are negatively charged and fluoresce orange. Spermatozoa with low membrane potential (depolarized) mitochondria are less negatively charged and fluoresce green.

The oxidative status of spermatozoa was assessed with the EasyKit 3 (ref. 025157; IMV Technologies). The kit contains red and green fluorochrome markers. Red fluorescence indicates spermatozoa with a damaged plasma membrane. The green fluorochrome penetrates the cell and emits green fluorescence upon oxidation, enabling an evaluation of the level of intracellular ROS.

### 2.5. Statistical Analysis

Statistical tests were conducted using SPSS Statistics for Windows, version 23 (IBM Corp., Armonk, NY, USA). Normal distribution tests were performed according to the Shapiro--Wilk test. Based on the normality test results, correlation tests between the variables were performed using the Pearson or Spearman correlation coefficient. The effect of cryopreservation on motility was assessed using paired sample t-test. Differences in lipid composition were examined using independent sample t-test or Kruskal–Wallis test. The means of pregnancy rate and the structural characteristics were compared by the Tukey test or the Games–Howell test. Data are presented as mean ± SEM. For all analyses, *p* < 0.05 was considered significant. 

## 3. Results

### 3.1. Part I: Spermatozoa Progressive Motility

#### 3.1.1. Progressive Motility and Physiological Parameters

A retrospective data analysis (*n* = 319 ejaculates from 58 bulls) was conducted to determine the PM of the spermatozoa (Figure 2). The maximum, median and minimum PM values were 84.77, 69.16 and 48.15%, respectively, with a mean ± standard deviation (SD) of 68.2 ± 7.85%. Based on this analysis, thresholds for high and low PM were defined (>74.0 and <62.5%, respectively).

Correlation analysis between PM and the spermatozoa’s physiological parameters revealed a strong positive correlation with the proportion of motile spermatozoa (r^2^ ≥ 0.994, *p* < 0.001) and spermatozoa with normal morphology (r^2^ ≥ 0.998, *p* < 0.001). A negative correlation was found between the concentration of spermatozoa ([M]/mL) and the proportion of progressively motile spermatozoa (r^2^ = 0.410, *p* < 0.001). No significant correlation was found between spermatozoa’s PM and velocity (r^2^ = 0.03, *p* = 0.5). 

#### 3.1.2. Fatty Acid Composition of Fresh Spermatozoa with High and Low PM

**Figure 3:**
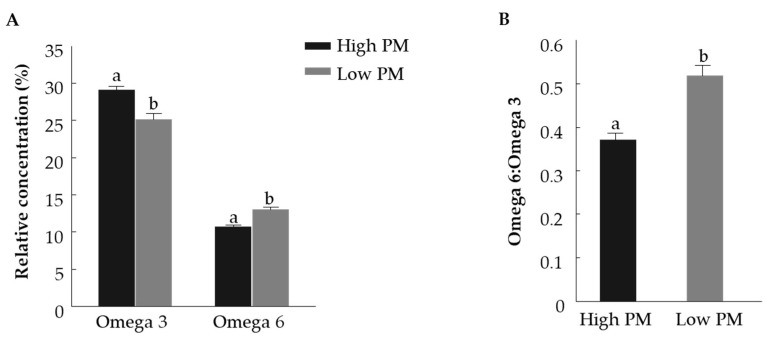
Relative concentrations of omega-3 and omega-6 polyunsaturated fatty acids (**A**) and omega-6 to omega-3 ratio (**B**) in fresh spermatozoa with high and low progressive motility (PM). Data are presented as mean ± SEM. Different letters above columns indicate significant differences between groups within omega-3 and omega-6 relative concentrations, separately, *p* < 0.005.

The fatty acid composition of fresh bull spermatozoa with high and low PM was examined (Table 1). The SFA concentration did not differ between the high- and low-PM groups. Similarly, the concentration of unsaturated fatty acids did not differ between the high- and low-PM groups. The proportions of linoleic acid (C18:2n6), adrenic acid (C22:4n6) and eicosadienoic acid (C20:2n6) were higher in spermatozoa of the low-PM vs. high-PM group (*p* < 0.01). The proportion of docosahexaenoic acid (DHA) (C22:6n3) was significantly higher in spermatozoa from the high-PM vs. low-PM group (*p* < 0.05). The proportion of omega-6 fatty acids was higher in the low-PM group than in the high-PM group (*p* < 0.01), (Figure 3A). The proportion of omega-3 fatty acids was higher in the high-PM group relative to the low-PM group (*p* < 0.01). Accordingly, the ratio of omega-6 to omega-3 fatty acids was higher in the low-PM vs. high-PM group (*p* < 0.01), (Figure 3B).

### 3.2. Part II: Progressively Motile Spermatozoa’s Survival

A retrospective data analysis (*n* = 3737 ejaculates from 133 bulls) was conducted to determine the cryosurvival of progressively motile spermatozoa. The maximum, median and minimum PMSS values were 89.7, 59.4 and 9.1%, respectively; the mean ± SD value was 58.0 ± 13.3%. The maximum, median and minimum PM values were 64.9, 65.4 and 38.4%, respectively; the mean ± SD value was 66.0 ± 8.1%. 

The proportion of motile spermatozoa following the freeze–thaw process was lower than in the fresh sample (*p* < 0.01; Table 2). The proportion of progressively motile spermatozoa also decreased following the freeze–thaw process and was lower than in the fresh sample (*p* < 0.01). Interestingly, the rate of decrease was statistically higher for total PM than for total motility (*p* < 0.01). Moreover, the rate of decrease was higher for spermatozoa with high PM vs. low PM (*p* < 0.01).

#### 3.2.1. Progressively Motile Spermatozoa’s Survival and Physiological Parameters 

The correlation analysis between ejaculate properties revealed a positive correlation between PMSS and dilution factor (r^2^ = 0.321, *p* < 0.001), spermatozoa’s velocity (µm/s) (r^2^ = 0.281, *p* < 0.001) and concentration ([M]/ml) (r^2^ = 0.162, *p* < 0.001). PMSS was negatively correlated with fresh spermatozoa’s motility (r^2^ = 0.261, *p* < 0.001).

#### 3.2.2. Fatty Acid Composition of High- and Low- PMSS for Fresh Spermatozoa

**Figure 4:**
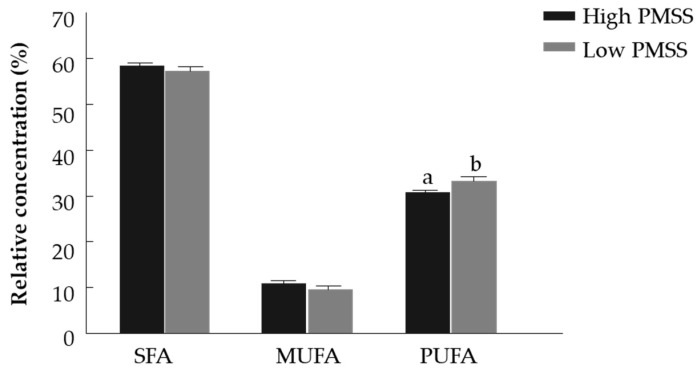
Relative concentration of saturated (SFA), monounsaturated (MUFA) and polyunsaturated (PUFA) fatty acids in fresh spermatozoa with high and low progressively motile spermatozoa’s survival (PMSS). Data are presented as mean ± SEM. Different letters above columns indicate significant differences between groups within PUFA relative concentrations at *p* < 0.005.

Fatty acid composition was examined in fresh spermatozoa collected from bulls with high and low PMSS (Table 3). The concentration of SFAs did not differ between the high- and low-PMSS groups. The proportion of myristic acid (c14:0) was higher in spermatozoa from the high-PMSS group (*p* < 0.05), whereas pentadecylic acid (c15:0), palmitic acid (c16:0) and arachidic acid (c20:0) were higher in those from the low-PMSS group (*p* < 0.01, *p* < 0.05 and *p* < 0.05, respectively). The concentration of stearic acid (c18:0) did not differ between the groups. The relative concentration of MUFAs did not differ between the high- and low-PMSS groups. The concentration of PUFAs was higher in the low-PMSS vs. high-PMSS group (*p* < 0.05), (Figure 4). The proportion of palmitoleic acid (c16:1n7) and linoleic acid (c18:2n6) was higher in the low-PMSS group (*p* < 0.01). The concentration of alpha-linolenic acid (c18:3n3), gamma-linolenic acid (c18:3n6) and eicosenoic acid (c20:1n9) was higher in the low-PMSS group (*p* < 0.05). The proportions of oleic acid (c18:1n9), arachidonic acid (C20:4n6), DHA (c22:6n3) and adrenic acid (c22:4n6) did not differ between the groups. The proportion of omega-6 fatty acids was higher in the low-PMSS group (*p* < 0.05). The proportion of omega-3 fatty acids did not differ between groups. The omega-6 to omega-3 ratio did not differ between low- and high-PMSS groups (Table 3).

#### 3.2.3. Structural Characteristics of Spermatozoa in the High- and Low-PMSS Groups

**Figure 5:**
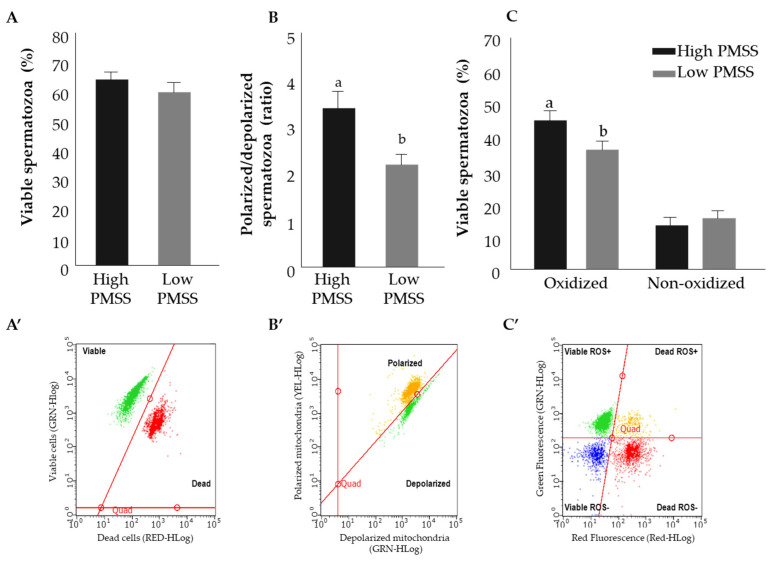
Structural characteristics of fresh spermatozoa in the high and low progressively motile spermatozoa’s survival (PMSS) groups. Presented are the proportion of viable spermatozoa out of total spermatozoa (**A**,**A’**, green); mitochondrial membrane potential expressed as the ratio of polarized to depolarized membrane (**B**,**B’**). Different letters above columns indicate significant differences between groups at *p* ≤ 0.005. The proportions of oxidized and nonoxidized viable spermatozoa are presented (**C**,**C’**). Data are presented as mean + SEM. Different letters above columns indicate significant differences between groups for oxidized viable spermatozoa at *p* ≤ 0.005. (**A’**–**C’**) are scatter plot representations of the results, expressed as a fluorescence intensity. GRN-HLog, YEL-HLog or RED-HLog indicate the green, yellow or red highest fluorescence intensity on log scale, respectively.

The structural characteristics of fresh spermatozoa in the high- and low-PMSS groups were examined (Figure 5). The proportion of viable spermatozoa did not differ between the groups. Mitochondrial membrane potential was higher in the high-PMSS vs. low-PMSS groups (*p* < 0.05). The proportion of viable cells with ROS expression was higher for spermatozoa in the high- vs. low-PMSS group (*p* < 0.05). The percent of viable spermatozoa, polarized-to-depolarized mitochondrial potential ratio and the percent of oxidized viable cells in frozen spermatozoa did not differ between high-PMSS and low-PMSS groups (52.7 vs. 50.2%, 2.2 vs. 2.8% and 37.1 vs. 38.8%, respectively).

### 3.3. Part III: Fertility Evaluation–Spermatozoa’s PM and In-Vivo Conception Rate

**Figure 6:**
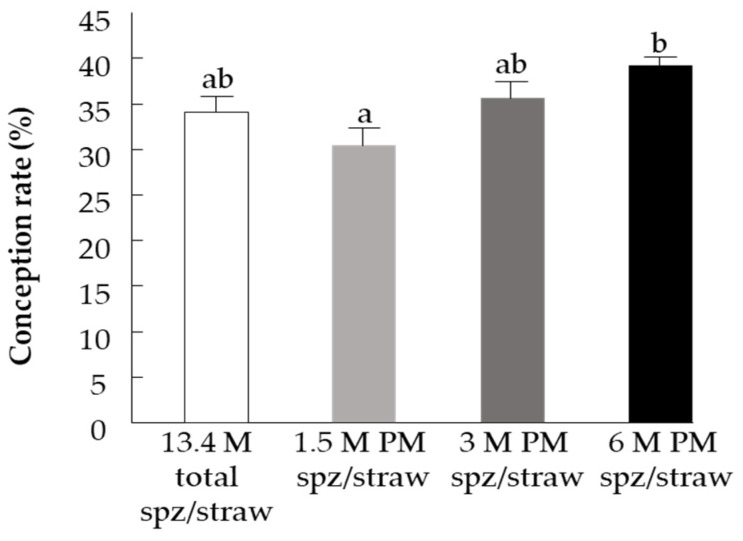
Pregnancy rate following insemination with straws containing 13.4 M spermatozoa (spz) per straw (control) or 1.5 M, 3 M, or 6 M progressively motile spermatozoa (PM spz) per straw. Data are presented as mean ± SEM. Different letters above columns indicate significant differences between groups at *p* ≤ 0.005.

The fertility study revealed a positive correlation between the number of progressively motile spermatozoa and conception rate (r^2^ = 0.379, *p* < 0.05). Further analysis revealed that the conception rate following insemination with 1.5 M progressively motile spermatozoa/straw was significantly lower than that using the straw containing 6 M progressively motile spermatozoa (*p* < 0.005), (Figure 6). In particular, there was a 14.6% increase in pregnancy rate when using straws with 6 M progressively motile spermatozoa and a 10.8% decrease in pregnancy rate when using 1.5 M progressively motile spermatozoa per straw, relative to the control.

## 4. Discussion

### 4.1. Progressive Motilty, Membrane Integrity and Fatty Acid Composition

Previous studies in boars [15], canines [16] and bulls [17] have associated membrane fatty acid composition with spermatozoa’s motility. In particular, membrane fatty acid composition directly affects the structure, physical properties and biological functions of the cell membrane associated with motility [22]. In the current study, we hypothesized that membrane fatty acid composition affects the spermatozoa’s PM, and that differences in fatty acid composition can explain, at least in part, the difference in spermatozoa with high and low PM. While the relative concentrations of saturated and unsaturated fatty acids did not differ between fresh spermatozoa with high vs. low PM, there were marked changes in the concentrations of omega-3 and omega-6 fatty acids between the groups. A high percentage of omega-6 was found in fresh spermatozoa with low PM, also reflected in a higher omega-6 to omega-3 ratio. These findings are consistent with previous work in humans, where a higher rate of omega-6 was found among men with asthenozoospermia/oligozoospermia, together with a higher omega-6:omega-3 ratio [23]. This ratio was also found to be higher in men with poor fertility and was negatively correlated with their spermatozoa’s motility and morphology [24].

One of the prominent differences in fatty acid composition was the relative concentration of DHA, which was higher in fresh spermatozoa with high PM compared to those with low PM. DHA accounts for about a quarter of the spermatozoa’s fatty acids and is a major PUFA in the spermatozoa membrane [17]. DHA is a long fatty acid with 22 carbons and 6 double bonds; this structure increases the spermatozoa membrane’s flexibility [25], which is necessary for tail bending and movement [26]. In humans, a positive correlation was found between spermatozoa’s motility and DHA concentration in the spermatozoa’s membrane [27]. A seasonal study in bulls reported high DHA levels in the tails of spermatozoa collected in the winter relative to those collected in the summer, associated with high PM [17]. 

Taken together, a lower omega-6 to omega-3 ratio and high levels of DHA in the membrane provide a possible explanation for high PM in fresh spermatozoa. 

Freezing-thawing is a stressful process which causes a membrane and cellular damage to spermatozoa [14]. Our findings suggest that variations in the fatty acid composition of spermatozoa with high PM vs. low PM underlie their cryotolerance (i.e., PMSS). It is suggested, therefore, that bulls will be further evaluated according to their PMSS, i.e., the ratio between the proportion of progressively motile spermatozoa after freeze–thawing and that in the fresh sample.

The relative concentrations of SFAs and MUFAs did not differ between high-PMSS and low-PMSS groups. However, PUFA concentration was higher in spermatozoa of the low-PMSS group. PUFAs readily undergo peroxidation due to the double bonds present in unsaturated fatty acids, and further oxidize proteins via secondary products (low-molecular-mass aldehydes such as acrolein, malondialdehyde and 4-hydroxynonenal aldehydes) [28]. This process disrupts the mitochondrial electron transport chain and promotes self-perpetuating mitochondrial ROS generation, causing additional oxidative stress [29]. Cryopreservation of bull spermatozoa has been reported to induce carbonylation (an irreversible oxidative modification) of proteins associated with energy metabolism and flagellum organization [28]. These processes might explain the higher sensitivity of spermatozoa with high PM to cryopreservation. The oxidative stress imposed during the freeze–thaw process can induce structural and functional modifications in the high PUFA content of the membrane, which in turn might reduce the spermatozoa’s ability to move straight forward.

In addition, examination of the membrane integrity of fresh spermatozoa revealed higher mitochondrial membrane potential and a higher proportion of viable cells expressing ROS in the high-PMSS group. The mitochondria provide accessible energy through ATP generation, which is transferred to the flagellum to drive motility [30]. Cryopreservation has been reported to decrease mitochondrial membrane potential in spermatozoa [31], possibly leading to reduced motility after thawing. Moreover, while mitochondria generates a low level of ROS during steady-state respiration [29], the cryopreservation process further increases the ROS level in bull spermatozoa [28]. ROS are required for basal cell functioning [29], but at a high level ROS can damage the cell. The spermatozoa have limited antioxidant capacity, making the ability to express ROS on one side and to stay viable on the other highly important for functioning. The high-PMSS group was expected to exhibit a better initial state for cryopreservation based on the membrane integrity evaluation of high- and low-PMSS groups found in the current study in fresh spermatozoa. However, post-thaw analysis did not show any differences in mitochondrial membrane potential or ROS level in spermatozoa of the high- vs. low-PMSS groups.

### 4.2. Progressive Motility and Cryopreservation of Spermatozoa

Cryopreservation of spermatozoa is an effective way of managing and preserving male fertility in both humans and domestic animals [32]. However, only about 60% of spermatozoa remain viable after the freeze–thaw process [33], with a significant decrease in PM from 60% in fresh spermatozoa to only 10 or 20% after thawing [34]. Here we report a disruptive effect of cryopreservation on both motility and PM of spermatozoa. Interestingly, spermatozoa with high PM were more sensitive to cryopreservation than their counterparts with low PM, expressed by a decrease in this parameter following the freeze–thaw process.

### 4.3. Progressive Motility and Fertility Competence

PM seems to be associated with semen fertilization ability, but data in the literature are scarce and inconclusive. Some studies have shown a significant positive correlation between bovine spermatozoa’s PM and fertility *in-vivo* [7] and *in-vitro* [8,9]. Other studies show that bulls with average or low fertility do not differ in their spermatozoa’s PM [35]. Narud et al. [36] reported that bulls with inferior fertility are characterized by high PM. Here we report a significant positive correlation between the number of progressively motile spermatozoa in a 0.225-mL straw and conception rate. The conception rate was highest when AI was performed with 6 M progressively motile spermatozoa per straw after thawing. Accordingly, analysis of physiological parameters indicated that PM positively correlates with motility and morphology, two features that are known to be associated with fertility potential [37]. This makes PM a promising parameter for predicting spermatozoa quality. Improving progressively motile spermatozoa’s survival during cryopreservation may enhance their fertilization potential.

## 5. Conclusions

This study provides additional support for PM as a parameter for evaluating the quality of spermatozoa. We showed that the number of progressively motile spermatozoa in a straw has a significant effect on conception rate. Differences between spermatozoa with high and low PM are most likely due to their membrane fatty acid composition, which also affects PMSS. Moreover, our findings indicate that the omega-6 to omega-3 ratio plays a prominent role in fresh semen PM, whereas PMSS is mainly affected by the total PUFA content, i.e., the number of double bonds in the membrane fatty acids rather than their positions. Association of sperm-quality parameters with PM and PMSS might provide a practical tool to predict spermatozoa’s quality. Our findings also suggest that cryopreservation differentially affects motility and PM with the latter being more sensitive. In particular, spermatozoa with high PM were found to be more sensitive to the cryopreservation process than those with low PM. This should be taken into account by AI centers during the process of straw preparation.

## Figures and Tables

**Table 1:** Fatty acid (FA) composition of fresh ejaculates with high progressive motility (high-PM; *n* = 9; >74.0%) and low progressive motility (low-PM; *n* = 6; <62.5%). Values are relative concentrations out of total fatty acids (mol %).

FA	High PM	Low PM	*p*-Value
C10:0	0.072	0.041	0.12
C11:0	0.068	0.071	0.92
C12:0	0.307	0.306	0.98
C13:0	0.053	0.041	0.27
C14:0	17.7	18.8	0.58
C15:0	0.2	0.2	0.99
C16:0	25.74	25.68	0.96
C17:0	0.279	0.263	0.54
C18:0	8.75	9.29	0.23
C20:0	0.169	0.211	0.06
C21:0	0.045	0.082	0.06
C22:0	0.200	0.219	0.47
C23:0	0.103	0.106	0.73
C24:0	0.384	0.248	0.35
C14:1	0.045	0.061	0.08
C15:1	0.218	0.255	0.07
C16:1n7 cis9	0.184	0.169	0.43
C18:1n7	2.79	2.98	0.23
C18:1n9 t	0.162	0.157	0.87
C18:1n9 cis	2.388	2.322	0.74
C18:2n6 cis9,12	4.28	5.726	<0.001
C18:3n3 cis12,15	0.114	0.131	0.19
C18:3n6	0.098	0.992	0.99
20:1n9	0.137	0. 139	0.87
C22:1 cis13	0.085	0.117	0.2
20:2n6	0.311	0.649	<0.001
20:3n6	0.757	0.730	0.69
C20:4n6	4.409	4.515	0.74
C20:5n3	0.043	0.047	0.65
C22:4n6	0.871	1.310	0.002
C22:6n3	29.01	24.93	0.03
SFA	54.086	55.642	0.389
MUFA	6.01	6.21	0.564
PUFA	39.9	38.1	0.346

SFA = saturated fatty acid; MUFA = monounsaturated fatty acid; PUFA = polyunsaturated fatty acid.

**Table 2:** Effect of cryopreservation on motility and progressive motility of bull spermatozoa.

Item		Fresh (Mean ± SD)	Frozen (Mean ± SD)	∆ (pp)
Motility (%)	Total	76.00 ± 9.69 ^a^	51.00 ± 7.04 ^b^	25.00 ± 11.19 ^X^
Progressive motility (%)	Total	66.00 ± 8.13 ^a^	37.81 ± 7.88 ^b^	28.19 ± 10.74 ^Y^
	High (>74.0%)	78.73 ± 3.31 ^a^	38.70 ± 8.32 ^b^	40.03 ± 8.96 *
	Low (<62.5%)	57.67 ± 3.75 ^a^	37.05 ± 7.51 ^b^	20.62 ± 7.88 **

∆ pp (percent points) indicates differences between fresh and frozen motility or progressive motility. SD = standard deviation. ^a,b^ Statistically significant difference within a row for fresh and frozen spermatozoa (*p* ≤ 0.001). ^X,Y^ Statistically significant difference within a column for total motility and progressive motility (*p* ≤ 0.001). *, ** Statistically significant difference for progressive motility subgroups (high and low) (*p* ≤ 0.001).

**Table 3:** Fatty acid (FA) composition of fresh ejaculates with high progressively motile spermatozoa’s survival (high-PMSS; *n* = 21; >68.1%) and low progressively motile spermatozoa’s survival (low-PMSS; *n* = 20; <54.6%). Values are relative concentration out of total fatty acids (mol %).

FA	High PMSS	Low PMSS	*p*-Value
C14:0	17.18	13.44	0.02
C15:0	0.28	0.35	0.01
C16:0	28.31	30.90	0.02
C18:0	12.35	12.10	0.61
C20:0	0.25	0.35	0.01
C16:1n7 cis9	0.26	0.33	0.005
C18:1n7	2.64	2.58	0.55
C18:1n9 t	0.41	0.45	0.34
C18:1n9 cis	7.32	5.93	0.12
C18:2n6 cis9,12	4.03	4.95	0.001
C18:3n3 cis 9,12,15	0.20	0.25	0.03
C18:3n6	0.23	0.37	0.001
20:1n9	0.25	0.32	0.02
20:3n6	0.59	0.55	0.84
C20:4n6	3.65	3.56	0.47
C22:4n6	0.97	1.01	0.81
C22:6n3	21.08	22.57	0.14
O3	21.28	22.82	0.118
O6	9.46	10.44	0.007
O6/O3	0.45	0.47	0.335

O3 = omega-3 FA; O6 = omega-6 FA; O6/O3 = ratio of relative concentrations of omega-6 to omega-3.

## Data Availability

The data sets used in this work are accessible from the corresponding author upon reasonable request.
